# Prevalence of sexual harassment among female medical staff in four Khartoum State tertiary hospitals

**DOI:** 10.11604/pamj.2023.45.30.36072

**Published:** 2023-05-15

**Authors:** Omer Osman Kheir, Hind Mukhtar Khair, Boladale Mapayi, Yashwi Haresh Patwa

**Affiliations:** 1 Research Department, National Center for Gastroenterology and Liver Disease, World Gastroenterology Organisation, Khartoum, Sudan,; 2 Obafemi Awolowo University Teaching Hospitals Complex, Ile-Ife, Nigeria

**Keywords:** Sexual harassment, female, psychological impact, prevalence

## Abstract

**Introduction:**

workplace harassment is hypothesized to cause a psychological impact on the welfare of the individual. The aim of this study is to estimate the prevalence of sexual harassment among female medical staff in Khartoum state tertiary hospitals.

**Methods:**

this is a cross-sectional hospital-based study in hospitals of Khartoum, Omdurman and North Khartoum. Our study population comprised female medical staff working in the outpatient clinics, emergency clinics, referred clinics and wards. The data was collected by online Google form self-administered questionnaire. The first part includes demographic information. The second part involved information regarding sexual harassment. The third part obtained information about the psychological status (effect) after harassment. The questionnaire was adopted and utilized from previous studies.

**Results:**

in this study, 325 participants were recruited. Among the participants, 51.4% were doctors, majority (81.2%) of the study population were single. The mean age was 26.29 and SD 3.865. Forty percent (40%) stated they had been sexually harassed at work. Forty-five percent (45%) reported the harassment occurred more than 3 times and 46.4% reported loss of desire for work. Action taken for investigation was only reported by 5.4%. The logistic regression revealed that female doctors were 1.45 times more prone to sexual harassment. Also, medical staff with low socioeconomic status were 83.2% chance not sexually harassed.

**Conclusion:**

the prevalence of sexual harassment among female medical staff was high. Doctors were more vulnerable to sexual harassment. And, the reported incidents were scarce.

## Introduction

Sexual harassment involves uninvited and undesired verbal or physical behavior of a sexual nature [1,2]. It is a form of gender discrimination that affects women and men in all areas of work. According to the International Labor Organization (ILO), sexual harassment can cause physical and psychological symptoms and illnesses as well as work-related consequences [3,4]. Moreover, those who experience sexual harassment respond to and cope with the harassment in a wide variety of ways [5]. Sexual harassment is a phenomenon rooted in cultures of injustice, hierarchical structures, and gender inequality and has occurred in all industries, including the healthcare industry [6]. The reactions of survivors to sexual harassment, suggest that there are four general types of responses: official reports, informal complaints, social support tactics, and attempts to communicate with the perpetrator [7].

A previous study conducted in the US among academic medical staff found that 30% of women stated they had experienced sexual violence in the workplace compared with only 4% of men [8]. A survey done among health workers in Rwanda showed that 39% had faced at least one form of workplace violence, such as verbal abuse, intimidation, and sexual harassment, over a year, with women disproportionately represented and affected in all categories [9]. In Kenya, community health workers report experiencing threats of violence from husbands of women they had allocated for HIV testing; upon which cases of rape were also reported, leading to calls for aid for security services to accompany community health workers [10].

Two studies have also found that most of the sexual harassment victims do not report the incidence [11,12]. There is limited data about the prevalence of sexual harassment among health workers due to stigma surrounding the sexual harassment and the low reporting rates. Therefore, the main aim of this study is to estimate the prevalence, common patterns and responses to sexual harassment among female healthcare workers in hospitals.

## Methods

**Study design and settings:** this was a cross-sectional study in hospitals of Khartoum, Omdurman and North Khartoum. There were 47 governmental hospitals in Khartoum state, which comprises 15 hospitals in Khartoum locality, 12 in Khartoum North locality and 20 hospitals in Omdurman locality. There are around 98 private hospitals in Khartoum state [13]. In this study, we included 3 governmental hospitals from each locality and 1 private hospital from Khartoum state. The sample was determined as 253 assuming an anticipated prevalence of 20.8% [14]. We used the design effect 1.5 to anticipate a non-response rate a total sample size was 384.

**Selection of study participants:** the sample was determined 384; 264 completed the online Google form from the 3 governmental hospitals and 43 participants refused to participate. Meanwhile, the remaining 77 were recruited it from a private hospital, 61 of them were cooperative and responded; meanwhile, while 16 were non-responsive. Our study population comprised medical staff working in outpatient clinics, emergency clinics, referred clinics (it is included in all medical specialties) and wards. Our inclusion criteria entailed participants aged 18 years old and above while the exclusion criteria were participants known to be psychologically impaired (psychological problem or disorder), on anti-depressant medications if not prescribed for sexual harassment trauma, or simply who refuse to participate. Participants were recruited between July 2020 and May 2021 at both private and governmental hospitals.

**Data collection procedure:** the data was collected by online Google form self-administered questionnaire (to ensure there is no missing data). The recruitment process was done by three senior house officers who approached the participants to fill in the Google form questionnaire in tablets. Before filling out the questionnaire, this definition was written down and explained. Sexual harassment occurs when someone acts in a sexually inappropriate manner that causes you to feel distressed, intimidated or offended. The questionnaire comprised three parts. The first part personified demographic information. The second part involved information regarding sexual harassment. The third part obtained information about the psychological status (effect) after harassment.

### Variables

***Dependent variables:*** exposure to sexual harassment.

***Independent variables:*** socio-demographic variables: age, marital status, residence, level of education, economic status, department, career duration.

***Other variables:*** forms of sexual harassment, number of exposures, place of occurrence, response after the harassment situation, psychological effects, who sexually harassed you, and action taken to investigate the causes of the sexual harassment. This questionnaire was adapted and utilized from previous studies [14,15]. The questionnaire comprised open-ended questionnaires. Multiple choice questions, some questions more than one response was allowed. Our sampling technique was nonprobability convenient sampling with a sample size of 325.

**Statistical analysis:** data was analyzed using SPSS version 24. Descriptive analysis was carried out by computing frequencies and percentages for categorical variables, and the mean and standard deviation for continuous variables. The chi-square test or Fisher´s exact test was used as appropriate to assess the difference between groups (relationship between sexual harassment at work and demographic variables). Univariate analysis for individual predictor variables of the outcome was conducted using logistic regression analysis.

**Ethical consideration:** ethical clearance was obtained from the Institutional Review Board (IRB) of Al-Neelen University. Written and verbal consent (we asked them if they will like to participate and fill out the questionnaire on the tablet) was obtained from all participants after an explanation of the questionnaire and objectives of the study. We maintained the complete confidentiality of data collected from participants. The responses of the subjects on the questionnaire were saved and locked in password-protected computers and were accessible only to the research team and principal investigator of the study. The participants were identified through a unique code recorded in the questionnaire, to provide the contributors with eminent psychological counseling afterward.

## Results

**Demographic characteristic:** a total of 325 participants responded to the Google form, making the response rate 84.6%. Among the participants, 51.4% were doctors, 16.6% were married and 81.2% of the study population were single. Other characteristics, (24%) of the respondents were working in outpatients. Forty-nine percent (49%) reported they were working day and night shifts. The mean age was 26.29 and standard deviation was 3.865. Regarding sexual harassment at work, 40% reported they had been sexually harassed. The economic level, residence, and education level (Table 1).

**Table 1:** the relationship between sexual harassment and demographic characteristics

Variable		Harassment at work		
		No count (%)	Yes count (%)	P-value*
Job	Doctorn167 (51.4%)	75 (38.5%)	92(70.8%)	<0.001
	Dentist 57 (17.5%)	36(18.5%)	21(16.2%)	
	Pharmacist 25(7.7%)	18(9.2%)	7(5.4%)	
	Lab tech 43 (13.2%)	42(21.5%)	1(0.8%)	
	Nurse 33 (10.2%)	24(12.3%)	9(6.9%)	
Marital status	Single 264 (81.2%)	152(77.9%)	112(86.2%)	P<0.045
	Married 54 (16.6%)	40(20.5%)	14(10.8%)	
	Divorced 7 (2.2%)	3(1.5%%)	4(3.0%)	
Economic status	Low less than $52 105(32.3%)	71(46.4%)	34(26.2%)	P<0.032
	Middle ($52-$150) 162(49.8%)	97(49.7.9%)	65(50.0%)	
	more than $150 58(17.8%)	27(13.8%)	31(23.8%)	
Residence	Khartoum 192 (59.1%)	119(61.0%)	73(56.2%)	P>0.658
	Khartoum North and Sharg al neel 77 (23.7%)	45(23.1%)	32(24.6%)	
	Omdurman 56(17.2%)	31(15.9%)	25(19.2%)	
Level of education	Bachelor 261 (80.3%)	149(76.4%)	112(86.2%)	P<0.011
	Masters 32(12.0%)	7(16.4%)	9(5.4%)	
	Doctoral 25(7.7%)	14(7.2%)	11(8.5%)	
Department	Dental 56(17.8%)	36(18.5%)	22(16.9%)	P<0.001
	Emergency room and intensive care unit 28 (8.6%)	12(6.2%)	16(12.3%)	
	Inpatient 62 (19.1%)	30(15.4%)	32(24.6%)	
	Laboratory 44 (13.5%)	42(21.5%)	2(1.5%)	
	Outpatient 78 (24.0%)	41(21.0%)	37(28.5%)	
	Pharmacy 25 (7.7%)	18(9.2%)	7(5.4%)	
	Surgery 30(9.2%)	16(8.2%)	14(10.8%)	
Working shift	Day only 152(46.8%)	106(54.4%)	46(35.4%)	P<0.001
	Night only 13 (4.0%)	11(5.6%)	2(1.5%)	
	Day and night 160 (49.2%)	78 (40.0%)	82 (63.1%)	

**Sexual harassment related information findings:** the most common type of harassment was unnecessary touch or unwelcome contact (22.3%) (Table 2). Forty-six participants reported sexual harassment occurred in a room (doctors´ room, nursing room, examination room). Forty-five percent (45%) reported harassments occur more than 3 times in the past 6 months. Participants reported 34.6% of the harasser were hospital staff/colleagues or supervisors (Table 3). The most common response after harassment, the response to this question, participants allowed to choose more than one response stated participants was ignoring the experience (13.1%), followed by recorded both fear and insecurity feeling and feeling of anger (11.5%), while those reported they ignored the experience and feeling of anger were 11.5% (Table 4). Of those who had been sexually harassed, 53% were psychologically affected. The most common psychological effect was the loss of desire for work (46.4%). Followed by fear and anxiety (21.7%). action taken for investigation was only reported by 5.4% (Table 3).

**Table 2:** types of sexual harassment

Types of sexual harassment	Frequency (%); n=130
Starring in a suggestive manner	14 (10.8%)
Making a sexual comment or jokes	6 (4.6%)
Talking by sexual words	5 (3.8%)
Verbal harassment	1 (0.8%)
Facing rape like situation	1 (0.8%)
Unnecessary touch or unwelcome contact	29 (22.3%)
Making a sexual comment or jokes, unnecessary touch or unwelcome contact	5 (3.8%)
Staring in suggestive manner, making a sexual comment or jokes	6 (4.6%)
Staring in suggestive manner, making a sexual comment or jokes, unnecessary touch or unwelcome contact	10 (7.7%)
Staring in suggestive manner, talking by sexual words	5 (3.8%)
Staring in suggestive manner, talking by sexual words, making a sexual comment or jokes	7 (5.4%)
Staring in suggestive manner, talking by sexual words, unnecessary touch or unwelcome contact	9 (6.9%)
Staring in suggestive manner, unnecessary touch or unwelcome contact	14 (10.8%)
Staring in suggestive manner, unnecessary touch or unwelcome contact, facing rape like situation	1 (0.8%)
Staring in suggestive manner, unnecessary touch or unwelcome contact, making hugs offer	1 (0.8%)
Talking by sexual words, making a sexual comment or jokes	4 (3.1%)
Talking by sexual words, making a sexual comment or jokes, making an intercourse offer	3 (2.3%)
Talking by sexual words, making a sexual comment or jokes, unnecessary touch or unwelcome contact	6 (4.6%)
Talking by sexual words, unnecessary touch or unwelcome contact	1 (0.8%)
Talking by sexual words, unnecessary touch or unwelcome contact, abusing authority for sex	2 (1.5%)

More than 1 response was allowed

**Table 3:** distribution of the sample according to place of occurrence, number of harassments, psychological effect, types of psychological effect, harasser, action taken for investigation and who took the action

Variable		Frequency (%)
**Place of occurrence; n=130**	Corridor	13 (10.0%)
	Room (doctor, nursing, examination)	61 (46.9%)
	Pharmacy	3 (2.3%)
	Lab	2 (1.5%)
	Reception	9 (6.9%)
	Stairs	5 (3.8%)
	Theatre	5 (3.8%)
	Corridor, stairs, elevator	9 (6.9%)
	Room (doctor, nursing, examination), corridor	3 (2.3%)
	Reception, room (doctor, nursing, examination ), corridor	20 (15.4%)
**Number of harassments; n=130**	Once	22 (16.9%)
	Twice	31 (23.8%)
	Three	18 (13.8%)
	More than 3	59 (45.4%)
**Psychological effect; n=130**	No	61 (46.9%)
	Yes	69 (53.1%)
**Type of psychological effect; n=69**	Anger	1 (1.4%)
	Ashamed	4 (5.8%)
	Depression	8 (11.6%)
	Disappointment	8 (11.6%)
	Fear and anxiety	15 (21.7%)
	Loss of desire to work	32 (46.4%)
	All the above	1 (1.4%)
**who harassed you? n=130**	Staff member/supervisor/colleague	45 (34.6%)
	Patient/co-patient/general public	43 (33.1%)
	Both	42 (32.3%)
**action taken for investigation; n=130**	No	123 (94.6%)
	Yes	7 (5.4%)
**Who take the action; n=7**	Community group	1 (14.3%)
	Management/employer	3 (42.9%)
	My self	1 (14.3%)
	Police	1 (14.3%)
	Union	1 (14.3%)

**Table 4:** distribution of the sample according to response after harassment

Response after the harassment	Frequency (%); n=130
Anger feeling	14 (10.8%)
Fear and insecurity feeling	6 (4.6%)
Ignore	17 (13.1%)
Shame and embarrassment feeling	4 (3.1%)
Told a friend/family	5 (3.8%)
feeling of anger, told a friend/family	11 (8.5%)
Fear and insecurity feeling, anger feeling	15 (11.5%)
Ignore, feeling of anger	15 (11.5%)
Shame and embarrassment feeling, fear and insecurity feeling	6 (4.6%)
Feeling of anger, left work place	7 (5.4%)
Shame and embarrassment feeling, anger feeling	4 (3.1%)
Shame and embarrassment feeling, left work place	6 (4.6%)
Feeling of anger, notified the relevant authorities	5 (3.8%)
Ignore, shame and embarrassment feeling	3 (2.3%)
Ignore, fear and insecurity feeling	5 (3.8%)
Fear and insecurity feeling, told a friend/family	7 (5.4%)

Participants were allowed to choose more than 1 response

**Demographic variables and sexual harassment:** the job category had a statistically significant relationship with sexual harassment (P<0.001), where doctors are more sexually harassed compared to others. Marital status was also significantly associated with sexual harassment (P<0.045), Also, respondents who were single reported more sexual harassment than their married, divorced or widowed counterparts, participants. Furthermore, the level of economic status was also statistically significantly associated with sexual harassment (P<0.032) (Table 1). The residence was not statistically significant with sexual harassment. The level of education was statistically significant with sexual harassment. Also, there was a statistically significant relationship between the department and sexual harassment. Moreover, there was an association between working shifts and sexual harassment. The logistic regression showed that only the job and the economic variables were associated with sexual harassment, doctors were 1.45 times more prone to sexual harassment compared to other categories and statistically significant (P<0.005). Also, those with low economic status (83.2%) had chances of not being sexually harassed, which is statistically significant (P<0.001); this model showed 68.9% predictability (Table 5).

**Table 5:** logistic regression according to sexual harassment

Variable	B	S.E.	Wald	P-value	Odd ratio	95% confidence interval for EXP(B)	
						Lower	Upper
**Age**	-0.068	0.048	1.970	0.160	0.934	0.850	1.027
**Job**			22.125	0.000			
Doctor	1.255	0.450	7.756	0.005	3.506	1.450	8.478
Dentist	0.539	0.531	1.030	0.310	1.714	0.605	4.851
Pharmacist	0.333	0.664	0.251	0.616	1.395	0.380	5.126
Lab technician	-2.632	1.096	5.769	0.016	0.072	0.008	0.616
**Marital status**	-0.019	0.340	0.003	0.956	0.981	0.504	1.911
**Economic status**			6.690	0.035			
Low less than 7000	-1.015	0.392	6.690	0.010	0.363	0.168	0.782
middle (7000-15000)	-0.633	0.352	3.245	0.072	0.531	0.267	1.057
**Residence**	0.218	0.165	1.738	0.187	1.244	0.899	1.720
**Level of education**	-0.086	0.254	0.115	0.734	0.917	0.557	1.510
**Working department**	-0.084	0.060	1.915	0.166	0.920	0.817	1.035
**Work shift**	0.089	0.151	0.350	0.554	1.093	0.813	1.470
**Constant**	1.430	1.379	1.074	0.300	4.177		

## Discussion

This study was one of the first studies that highlighted the prevalence of sexual harassment among female medical staff in Sudan. The prevalence of sexual harassment reported by Sudanese female medical staff was lower than the one reported in Egypt [15] and Nigeria [16]. However, it is higher than the one conducted in the US [8] This may be related to the law and legislation that protects against sexual harassment. According to a report conducted in 2018 by the World Bank legislation, which examined specific legislation that protects against sexual harassment in employment and education with criminal penalties attached to those legislations in 189 countries; showed that nearly 60 countries had no law that protects against sexual harassment. It also stated that 70 economies in the Middle East and North Africa have zero legislation. A 2018 report from National Academies of Sciences, Engineering, and Medicine (NASEM) indicates that job stress and mental/physical health consequences of workplace sexual harassment are linked to declines in employee performance, advancement, and retention [17]. Previous study, indicated that sexual harassment affects the well-being status of targets and may result in physical, mental, behavioral, and social consequences, depending on the events; these events may be serious and long-lasting [18].

In our study, preparators were more likely hospital staff/colleague/ supervisors were the perpetrators followed by co-patients which come into agreement with the Nigerian study [16]. The most common type of harassment was needless touch or unwelcome contact followed by staring in a suggestive manner which coincides with previous study conducted by Turkey [14]. The most common place for sexual harassment was reported in a room (doctor´s room, nursing room, examination room); as it escalates the chances for the victim to be alone and perpetrators can easily start the harassment, and no one will interfere.

In this study, more than fifty percent of those who had been sexually harassed were psychologically affected. The most common psychological effect was loss of desire for work. Followed by fear and anxiety. Previous studies reported association of workplace sexual harassment on employee's work performance, mental health, and loss of job interest, which may potentially lead to chronic anxiety and loss of employment [19-22]. Multiple harassment occurrences were reported in this study. which can be explained due to low incident reporting by the victims. Also, the hostile environment may have a role to play and investigation was only reported by 5.4%. According to a study carried out in Australia and South Wales in the years 2015 and 2016, only 16-19% reported harassment and it showed it was ineffective and personally harmful when informing a senior colleague, which coincides with our study that a few reported harassments out of fear and defame especially when the victim was single. Yet according to a study carried out in India in 2007, it showed females kept quite out of fear of social stigma and it was difficult to get married afterwards [23,24]. Sexual harassment was found to be greater among single women and was statistically significant compared to married and divorced women; with regard to family status, single females tend to be targeted more than married females (De Coster *et al*. 1999; Ragins and Scandura 1995). De Coster and colleagues (1999) theorize that single females are perceived as challenging traditional family structures and are viewed as less protected and more sexually available [25].

**Study limitations**: one of these limitations is that the sample was a convenient sample. Therefore, this limited the external validity and generalization of the results. Another limitation is the study participants. Level of nonresponse is one concern, but a greater one still is that of biased response, we suggest cultural influence may play a role in participants’ refused to participate. The study design made it possible to obtain information on sexual harassment from medical staff working in hospitals in Khartoum. This data was not available beforehand. The reliability test of the questionnaire was not performed and could be considered as a potential limitation.

Study findings revealed the alarming need to report to the highest authority and to take this injustice with critical consideration; to address this problem and necessary actions taken to abolish these accidents.

## Conclusion

To conclude, sexual harassment was high among female medical staff. The most common culprits were doctors or colleagues. Most of those victims had loss of desire of work and reports of those incidents were rare. Therefore, we suggest to create a movement such as “Me too movement” to raise awareness of the problem for the betterment of the society.

### What is known about this topic

- According to our knowledge there is no available data about sexual harassment among female medical staff in Khartoum.

### What this study adds

- Contribute valuable information to the currently limited literature documenting the current sexual harassment against female medical staff and the types and psychological effect of sexual harassment (About forty percent of sexual harassment was reported).
